# Evaluating the accuracy and biological meaning of visits to RFID‐enabled bird feeders using video

**DOI:** 10.1002/ece3.8352

**Published:** 2021-11-16

**Authors:** Eric J. Hughes, Rachael P. Mady, David N. Bonter

**Affiliations:** ^1^ Department of Natural Resources & The Environment Cornell University Ithaca New York USA; ^2^ Cornell Lab of Ornithology Ithaca New York USA

**Keywords:** bird feeding, data validation, GoPro, passive integrated transponder tags, radio‐frequency identification, supplemental food, video, visit duration

## Abstract

Radio‐frequency identification (RFID) technology has gained popularity in ornithological studies as a way to collect large quantities of data to answer specific biological questions, but few published studies report methodologies used for validating the accuracy of RFID data. Further, connections between the RFID data and the behaviors of interest in a study are not always clearly established. These methodological deficiencies may seriously impact a study's results and subsequent interpretation. We built RFID‐equipped bird feeders and mounted them at three sites in Tompkins County, New York. We deployed passive integrated transponder tags on black‐capped chickadees, tufted titmice, and white‐breasted nuthatches and used a GoPro video camera to record the three tagged species at the feeders. We then reviewed the video to determine the accuracy of the RFID reader and understand the birds’ behavior at the feeders. We found that our RFID system recorded only 34.2% of all visits by tagged birds (*n* = 237) and that RFID detection increased with the length of a visit. We also found that our three tagged species and two other species that visited the feeders, American goldfinch and hairy woodpecker, retrieved food in 79.5% of their visits. Chickadees, titmice, nuthatches, and woodpeckers spent, on average, 2.3 s at feeders to collect one seed per visit. In contrast, goldfinches spent an average of 9.0 s at feeders and consumed up to 30 seeds per visit. Our results demonstrate the importance of confirming detection accuracy and that video can be used to identify behavioral characteristics associated with an RFID reader's detections. This simple**—**yet time‐intensive**—**method for assessing the accuracy and biological meaning of RFID data is useful for ornithological studies but can be used in research focusing on various taxa and study systems.

## Graphical abstract

Radio‐frequency identification (RFID) technology has gained popularity in ornithological studies as a way to collect large quantities of data to answer specific biological questions, but relatively few published studies report methodologies used for validating the accuracy of RFID data. Further, connections between the RFID data and the behaviors of interest in a study are not always clearly established. Here, we describe a simple and inexpensive method for determining the accuracy and biological meaning of RFID data.

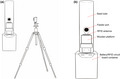

## 1 INTRODUCTION

The development and integration of increasingly advanced technologies into ecological research has expanded researchers' data collection and analysis capacity (Allan et al., 2018; Marvin et al., 2016). Researchers can now uniquely tag organisms and use automated detection systems to acquire large amounts of data that would otherwise be unattainable through conventional approaches (Ropert‐Coudert & Wilson, 2005). In particular, for projects involving field work, many hours may be spent navigating to and from study sites as well as on‐site collecting data. Technology that can make “observations” in place of a human can be extremely valuable, especially when that technology can record data continuously or under weather/site conditions that make data collection challenging for human observers. Additionally, using technology in place of human observers can avoid any influences that human presence may have on study organisms and their ecosystems (Riley & Bezanson, 2018).

While the use of technology can be an asset in ecological research, it is imperative to recognize potential drawbacks or limitations associated with each technological solution as well as identify and measure potential sources of error or bias. Such constraints on technology use include high equipment cost (Greenville & Emery, 2016), data storage capability (Pimm et al., 2015), and variation in the accuracy of data being collected (Wu & Hobbs, 2002). While human error can be problematic for data collection efforts, technology may also produce “bad” data that are ultimately unsuitable for answering the intended research question (Brown et al., 2018).

Radio‐frequency identification (RFID) is one type of technology that can collect large quantities of data with limited human involvement. An RFID system is composed of a reader and transponders; the reader records the unique identifying code of each transponder that passes within range of its antenna. RFID technology has a wide variety of applications, including building access and security (Farooq et al., 2014), healthcare (Kranzfelder et al., 2012), electronic toll collection (Satyasrikanth et al., 2016), supply chain management (Ampatzidis & Vougioukas, 2009; Oghazi et al., 2018), livestock monitoring (Brown‐Brandl et al., 2019; Maselyne et al., 2014), and poultry monitoring (Sales et al., 2015). In the natural sciences, RFID has been used in studies involving a wide range of taxa including fish (Fetherman et al., 2014), arthropods (Batsleer et al., 2020; Nunes‐Silva et al., 2019), mammals (König et al., 2015), and birds (Bonter & Bridge, 2011; Ferreira et al., 2020).

For avian research, RFID systems have become increasingly affordable and accessible, allowing researchers to answer research questions previously thought impossible (Bridge & Bonter, 2011). Passive integrated transponder (PIT) tags, a type of RFID tag, do not require an onboard power source and are consequently small and lightweight (often <0.5 g, making attachment to small birds possible). In ornithological research, transponders are attached to individuals either subcutaneously or as an external tag, and the antenna of the RFID reader is oriented so that the tagged individuals must move directly over or through it to be detected (Bonter & Bridge, 2011). When PIT tags come within range of an RFID antenna's electromagnetic field, they are energized and broadcast their identifying code to the reader. In the context of ornithological research, communication between the tag and the reader typically occurs at a low frequency (Subramanian et al., 2005). Low‐frequency PIT tags have a restricted detection range and need to come within approximately 30 cm of an antenna to be detected (Bonter & Bridge, 2011). That detection range varies based on the frequency range of the system, positioning and spatial relationship between transponders and the antenna, and the shape and size of the antenna.

Despite the rapidly increasing use of RFID technology in avian ecology, few published studies report methodologies used for validating the accuracy of the data (but see Iserbyt et al., 2018), and there is no established standard for RFID data validation. Technology may not perform as expected, and accuracy assessments are needed to determine the utility of a technology for recording meaningful information under different research conditions or with different taxa. The accuracy of RFID detections varies across designs and applications of the technology (Firth et al., 2018; Zárybnická et al., 2016), and missed detections are a potential source of error and bias. With limited read ranges, the PIT tags used with birds require tagged individuals to move over a particular location, something that could be difficult to achieve without modifying natural behaviors. Many birds, especially small passerines, are fast‐moving and may pass over or through the antenna before the reader can detect the individual's tag. Detection rates of 100% are not always expected nor are they universally necessary; however, low detection accuracy could lead to biased data and incorrect conclusions. Moreover, quantification of detection rates can aid interpretation of the data or modification of the RFID design.

Validating RFID data is not only key for forming sound conclusions but also important for understanding the biological relevance of collected information. RFID data can link an individual to a fixed location at a specific point in time, but that information alone may not capture the behavior of interest. For example, a PIT tag being detected by the RFID system at a bird feeder informs the researcher that the bird was at the location, but it does not indicate whether the bird successfully foraged. Further, an RFID detection for one species may not have the same biological meaning as a detection for another species. Consider the variable foraging strategies of common feeder birds: some, such as chickadees and titmice (Paridae), nuthatches (Sittidae), and some woodpeckers (Picidae), visit feeders momentarily to take one seed to open elsewhere (Erlwein, 1996; Mumme, 2003), while others, such as finches (Fringillidae), consume many seeds during a single feeder visit (Horn, 1995). It is imperative that researchers understand the behavior that is being captured in RFID detections for each species being studied. RFID technologies are often deployed to answer specific biological questions, yet the connections between the RFID data and the behaviors of interest are not always clearly established. These connections often need to be calibrated via in‐person observation or by analyzing videos of tagged individuals as they interact with the RFID system (Bonter & Bridge, 2011; Nomano et al., 2014).

The objectives of this study were to use video analysis to (1) assess the accuracy of RFID‐enabled bird feeders in recording visits by birds and (2) assess the biological meaning of a visit to a bird feeder by quantifying visit length and foraging success rates. We paired video recordings with detections from the RFID‐enabled bird feeders to quantify the accuracy of the RFID readers in recording the visitations of species that regularly visit feeders. We tested for correlations between successful detections by the RFID system and three PIT tagged species (black‐capped chickadee *Poecile atricapillus*, tufted titmouse *Baeolophus bicolor*, and white‐breasted nuthatch *Sitta carolinensis*), study site, and duration of visit, as well as correlations between successful foraging by individual birds at the supplemental feeder and the same three variables. Additionally, we tested for correlations between duration of a visit and the five species that visited the feeders most frequently (American goldfinch *Spinus tristis*, black‐capped chickadee, hairy woodpecker *Leuconotopicus villosus*, tufted titmouse, and white‐breasted nuthatch) to demonstrate how a detection by an RFID reader may have different biological meanings for different taxa.

## 2 MATERIALS AND METHODS

Fieldwork was conducted at three sites in Tompkins County, NY, USA (42.443°, −76.501°). The feeder sites were located between 4.5 and 8.2 km apart and were selected based on criteria used by Mady et al. (2021). Specifically, each site was located within a stand of mixed deciduous‐coniferous forest with open understory, similar land use history and forest age, and only a small number of nearby private homes (where supplemental food may be offered).

**FIGURE 1:**
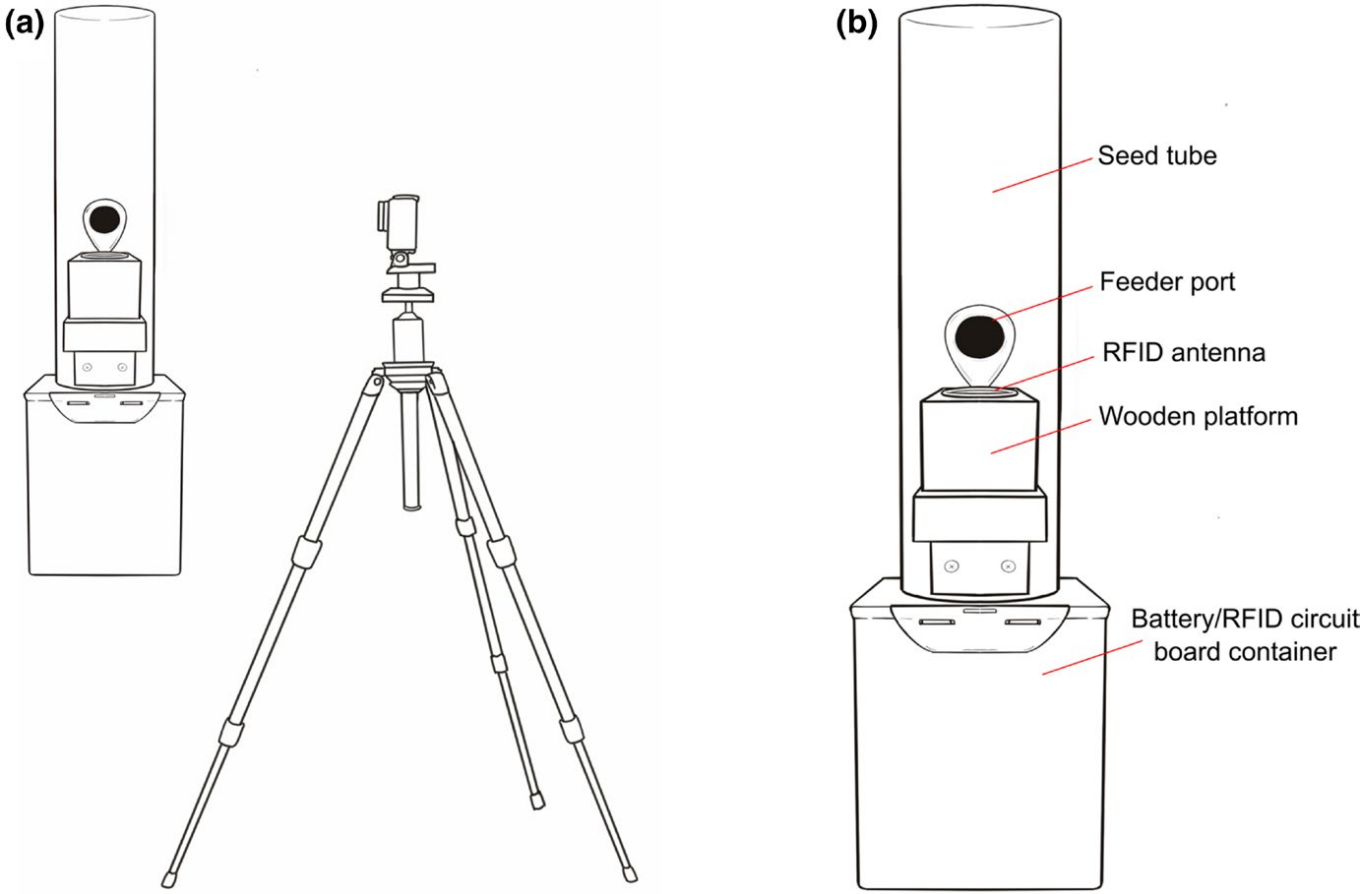
Illustration depicting the orientation of the tripod‐mounted GoPro video camera relative to a radio‐frequency identification (RFID)‐equipped bird feeder (a) and a magnified view of the feeder highlighting its key components (b). Graphic by Jillian Ditner

We installed and filled one feeder at each of our three field sites with black‐oil sunflower seed in August 2018 and used mist nets to capture black‐capped chickadees (*n* = 37), tufted titmice (*n* = 17), and white‐breasted nuthatches (*n* = 13) from August to November 2018. The birds were fitted with one aluminum United States Geological Survey (USGS) leg band and a unique combination of one colored plastic leg band and one colored leg band with an integrated PIT tag (EM4102, 125 KHz, Eccel Technology Ltd, Aylesbury, UK). We designed the feeders following Bridge and Bonter (2011) with a few modifications. First, we ordered copper‐wire antennas (outer diameter: 10.2 cm, 125 kHz; QKits Electronics, Kingston, Ontario), which we unraveled until they achieved the optimal inductance of 1.35 mH (Bridge & Bonter, 2011). We then coated the wire in black rubber (Plasti Dip, Blaine, Minnesota). Then we attached each antenna with Gorilla tape (The Gorilla Glue Company, Cincinnati, Ohio) to a wooden platform mounted on the feeder tube under the feeder port (Figure 1). We used a wooden platform instead of the two‐dowel design described by Bridge and Bonter because we believed the platform would offer better support for the antenna and facilitate easier use of the feeders by our focal species. We provided only one feeding port (diameter: 2.2 cm) per feeder to maximize detection probability by requiring tagged birds to perch on the platform with the RFID antenna in order to access food.

The antenna was connected to and powered by RFID circuit boards that were designed and created by Bridge and Bonter (2011). We set the circuit boards to have a poll time of 500 ms, cycle time of 1 s, and delay time (minimum period of time between successive tag recordings) of 1 s. Boards were powered by 12 or 14.4 V batteries. We set the battery threshold to 9 V to avoid deterioration of the read quality if the batteries ran low. We replaced batteries every 2–7 days and housed the battery and the circuit board in a waterproof container attached to the bottom of the PVC feeder tube. Each time we changed the system's batteries, we confirmed that the RFID reader was recording tags properly by downloading the data and holding a test PIT tag over the antenna to simulate a visit. Had the test tag not been recorded, we would have changed the system's antenna; however, the test tag was always registered by the reader during data collection.

We filled the three RFID‐enabled feeders such that food was continuously available. In February and March 2019, we placed a GoPro HERO3 video camera (GoPro, San Mateo, California) mounted on a pole approximately 20 cm from the feeder at each site to record visits by individual birds. We positioned the camera to record visiting birds from the side of the feeding port, allowing us to easily see whether an individual took food during each visit and to visually record the leg band combinations. We installed the video camera at each feeder for 2.5 h once a week for 6 weeks and randomized the order in which feeders were recorded. The 2.5‐h recording periods began as early as 9:30.a.m. and as late as 2:30 p.m.

The GoPro automatically split each day's recording into 13‐min segments. For video analysis, one observer (EJH) randomly selected and subsequently analyzed six video segments (totaling 78 min of video) from each of the three sites. The six video segments selected for each site spanned 3–4 days of the six total days of recording, and up to three video segments were sampled from each day. To minimize the potential impacts of human presence on visitation behavior, we eliminated the first and last video segments from each day when the camera was being installed or retrieved. We also intended to mitigate potential effects of neophobia, or fear demonstrated in response to novel stimuli (Greenberg, 2003), by eliminating the first video segment. EJH recorded the time of individual visits, and the species and identity (if banded) of the visiting bird. For each visit, EJH also recorded the visit duration (rounded to the nearest whole second), and whether or not the visit resulted in the bird leaving with a seed.

To quantify the “success rate” of the RFID system in detecting visits of banded birds, we compared the visits observed in each analyzed 13‐min video segment with those recorded by the RFID system over the same 13‐min range. Matching visits were coded as a successful RFID detection, whereas a visit recorded on video that was not recorded by the RFID unit was coded as a failed detection.

### 2.1 Statistical analyses

To test for factors associated with the likelihood of a bird being detected by the RFID system, we modeled RFID detection (Yes = 1/No = 0) as a function of visit length, species (chickadee, nuthatch, or titmouse), and site (1, 2, or 3) using a generalized linear mixed model with a binomial error distribution. We also included individual identity as a random effect because the same individuals could visit a feeder multiple times, and a quadratic term for visit length because we expected RFID detection to have a nonlinear relationship with visit length. Additionally, we included an interaction between species and visit length because we expected the effect of visit length on RFID detection could differ by species because of differences in morphology (e.g., leg size) or behavior. Using likelihood ratio tests, we found that the model including visit length as a quadratic predictor did not perform better than the model with visit length as a linear predictor (*X* ^2^_1_ = 0.389, *p* = .533). Additionally, the data did not support the model with the interaction between species and visit length (*X* ^2^_1_ = 2.027, *p* = .363). Thus, we report on the results from the RFID detection model with visit length, species, and site as linear predictors with individual ID as a random variable.

Additionally, we explored the magnitude of variability in the detection rates among individual RFID tag–bird combinations. We calculated the model‐based predicted probabilities of detection by holding visit length to its mean value (1.87 s) and site as the site with the “middle” predicted probability of RFID detection (site 2; the site where the random‐effect coefficient was closest to zero). Predicted values were calculated on the logit‐scale values and then we back‐transformed the estimates so they could be graphed as probabilities.

To test for factors associated with the likelihood of successfully acquiring seed, we modeled foraging success (Yes = 1/No = 0) as a function of visit length, species (tagged: black‐capped chickadee, tufted titmouse, or white‐breasted nuthatch), and site using a generalized linear mixed model with a binomial error distribution. As with the previous model, we included a quadratic term for visit length because we expected foraging success to have a nonlinear relationship with visit length, and individual ID as a random effect to account for individual variation. Using likelihood ratio tests, we found that the model including visit length as a quadratic predictor did not perform better than the model with visit length as a linear predictor (*X* ^2^_1_ = 0.027, *p* = .869), so we report on the results from the model with visit length as a linear predictor.

To determine if the length of a visit at a feeder varied among species, we modeled visit length as a function of species and site using a generalized linear model with a zero‐truncated negative binomial distribution. We used a zero‐truncated distribution because there could not be any visit lengths of 0 s. We included any visits by individuals of species, banded or unbanded, that had a minimum of 50 visits recorded across all sampling windows and all three feeders combined. These species included white‐breasted nuthatch, black‐capped chickadee, tufted titmouse, American goldfinch, and hairy woodpecker.

We conducted statistical analyses in R version 4.0.0 (R Core Team, 2020) using generalized linear models with the *glmmTMB* package (Brooks et al., 2017). Of the total 237 visits by tagged individuals, we excluded three visits that were greater than 6 s long because of the small sample size of longer visits. Of the total 667 visits by tagged and untagged individuals, we excluded visits that exceeded 100 s (*n* = 2) because the video indicated that the individuals were perched on the feeder, not feeding or moving, and most likely avoiding detection by a nearby predator (Caro, 2005). We confirmed that model assumptions were met for each model using a simulation‐based approach with the *DHARMa* package (Hartig, 2020). We assessed the overall effect of each predictor variable with Type III Wald‐*χ* ^2^ tests using the *car* package (Fox & Weisberg, 2019).

To better understand the biological relevance of the model results, we used the *emmeans* package (Lenth, 2021), to calculate the estimated marginal means (EMMs) and predicted probabilities for categorical predictor variables as well as to assess the statistical significance (*p* ≤ .05) of their pairwise differences. We calculated model predictions for continuous predictors on the response scale using the *ggeffects* package (Lüdecke, 2018). All results were plotted using the *ggplot2* package (Wickham, 2016).

## 3 RESULTS

EJH watched 78 min of video for each site sampled over 3–4 days of recording, tallying 667 visits by birds to the focal feeders. PIT tagged chickadees, titmice, and nuthatches were responsible for 237 of the 667 visits (35.5%), and 114 of the 237 visits by tagged birds were chickadees, 70 were titmice, and 44 were nuthatches. During the sampling periods, 22 of 37 PIT tagged chickadees (59.5%), 11 of 18 PIT tagged titmice (61.1%), and 7 of 13 PIT tagged nuthatches (53.8%) at the study sites visited the feeders. The number of visitations by individuals of each species varied, with chickadees visiting between 1 and 12 times, titmice visiting between 1 and 14 times, and nuthatches visiting between 1 and 19 times.

### 3.1 RFID performance

**FIGURE 2:**
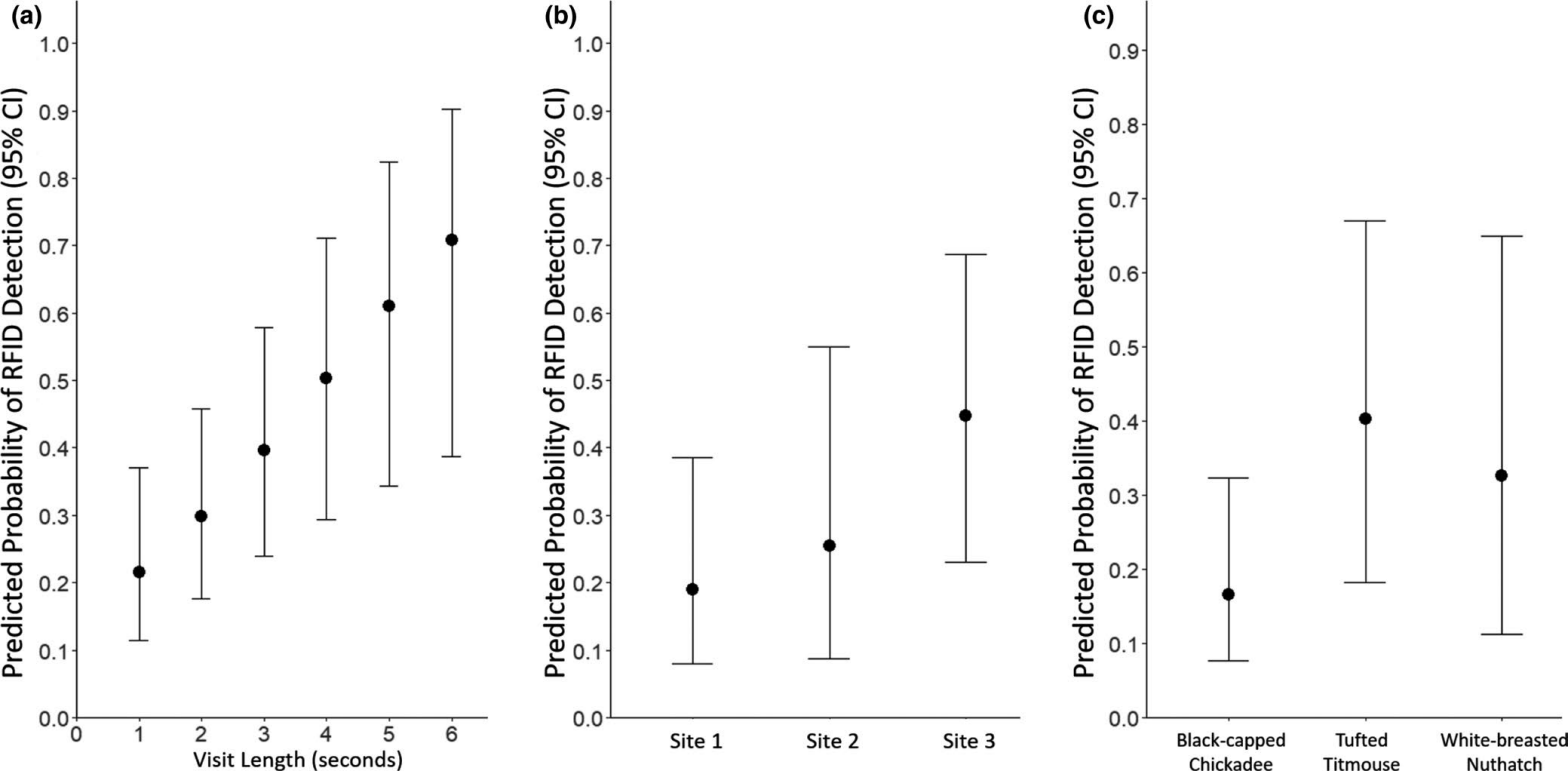
The average predicted probability of the radio‐frequency identification (RFID) system detecting a bird visiting the feeder by (a) visit length, (b) site, and (c) species with 95% confidence intervals

**FIGURE 3:**
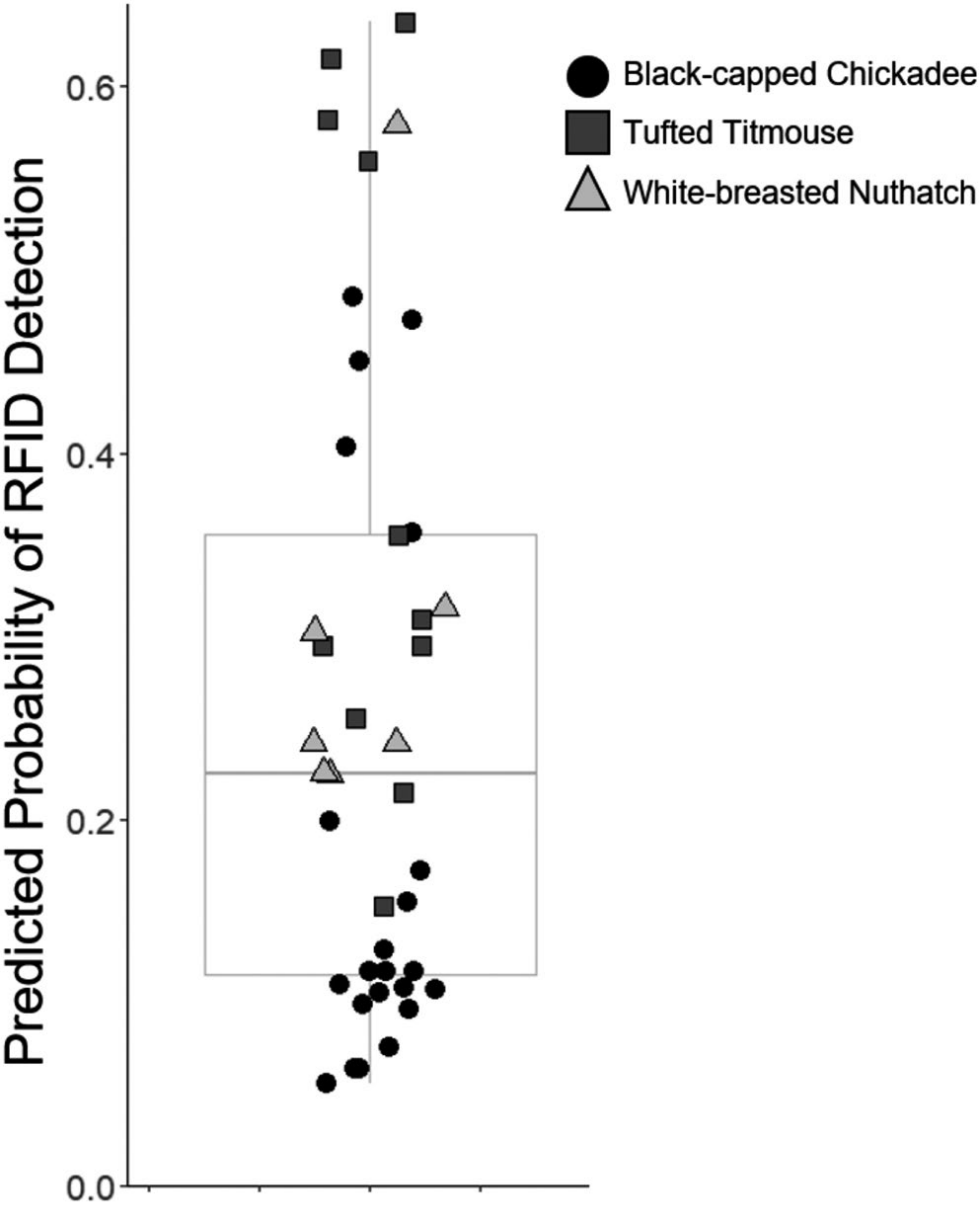
The estimated probability of radio‐frequency identification (RFID) detection varied among individual birds. Each point represents the estimated probability of an individual's visit being detected, holding constant all fixed effects (visit length =1.87 s, site =2) except for species; the estimated probabilities were calculated for the species that was associated with each RFID tag. The species of each point is referenced by color and shape (black circle = black‐capped chickadee, dark gray square = tufted titmouse, and light gray triangle = white‐breasted nuthatch). Symbols are spread across the horizontal axis to reduce overlap and improve legibility

Overall, 34.2% of the 237 feeder visits by PIT‐tagged birds were recorded by the RFID system. The likelihood of RFID detection increased with visit length (*X* ^2^_1_ = 8.464, *p* = .004), but did not vary by species (*X* ^2^_2_ = 3.624, *p* = .163) or across sites (*X* ^2^_2_ = 3.362, *p* = .186, Figure 2). In exploring variation in the probability that RFID tags were detected, we found that 75% of the predicted individual detection probabilities were ≤0.36, with considerable variation among individuals (range of mean predicted probabilities: 0.06 to 0.64, Figure 3). Of the 237 visits by tagged birds, 181 (76.4%) resulted in at least one seed being taken, while 56 (23.6%) resulted in the bird leaving without a seed. The RFID system detected 78 of the 181 “successful” visits (43.1%) and only 3 of the 56 visits without any seeds taken (5.4%).

### 3.2 Characteristics of a visit

**FIGURE 4:**
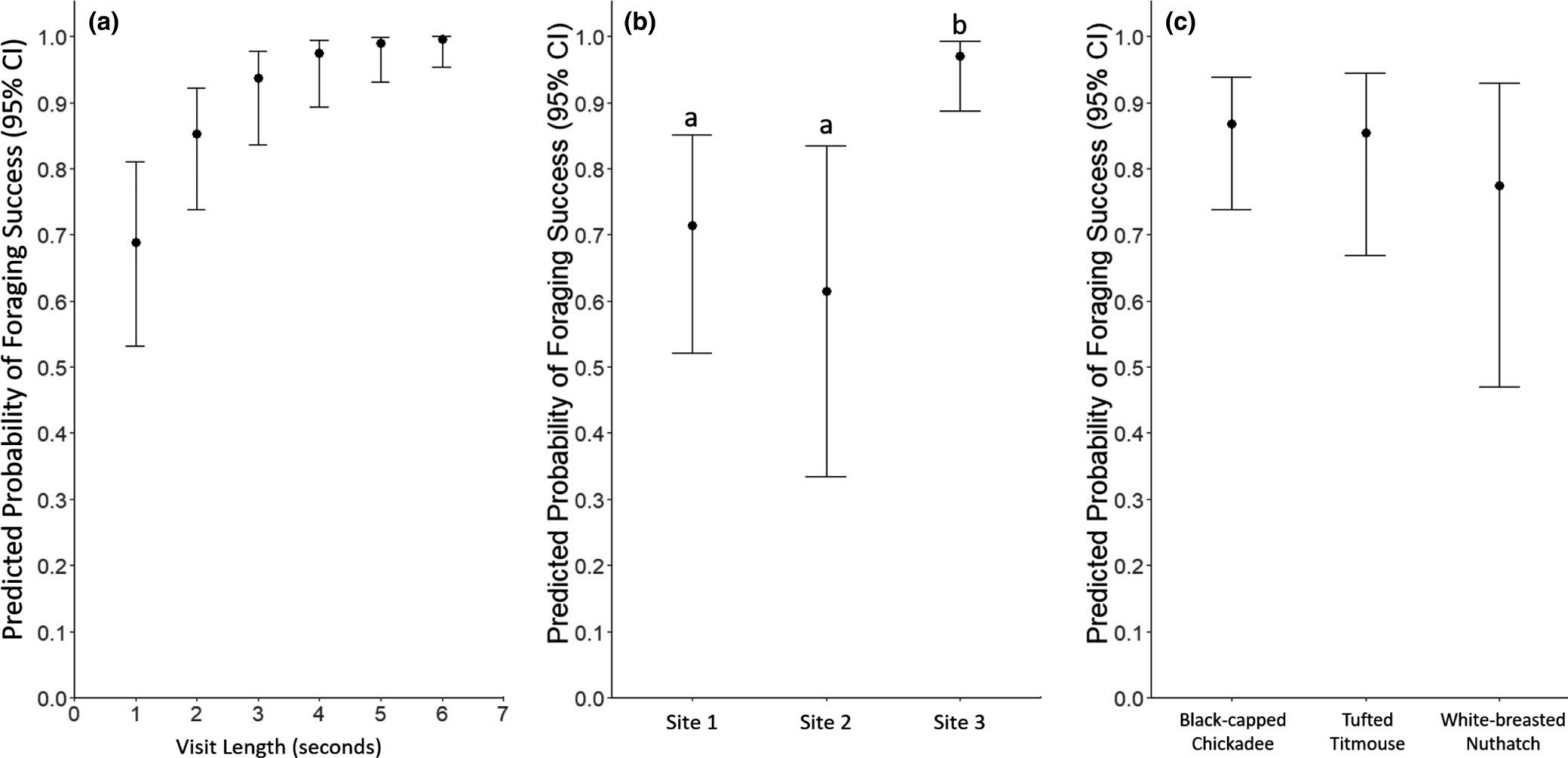
The average predicted probability of successfully acquiring a seed (foraging success) by (a) visit length, (b) site, and (c) species with 95% confidence intervals. Visit length and site were statistically significant predictors in the model, and in panel (b), letters indicate a statistically significant (*p* ≤ .05) difference in a post‐hoc pairwise comparison between sites on the log‐scale

Of the 667 total visits by our five focal species, 530 visits resulted in a bird either consuming a seed at the feeder or leaving with a seed (mean = 1.17 seeds ± 0.06 standard error when at least 1 seed was taken). When consuming seeds at the feeder, individuals took 1–30 seeds (Table 1). Conversely, when leaving the feeder with seeds, only 1–2 seeds were taken. While foraging success did not vary among the three tagged species (*X* ^2^_2_ = 0.6751, *p* = .714), it did vary by visit length and site (*X* ^2^_1_ = 12.530, *p* = .026; *X* ^2^_2_ = 11.740, *p* = .003). The average predicted probability of foraging success increased with visit length and was higher at one site compared with the other two (Figure 4).

**TABLE 1:** Characteristics of feeder visits by each focal species

Species	Number of visits	Average (± standard error) number of seeds taken or consumed per visit	Range of seeds taken or consumed per visit
Hairy woodpecker	74	0.93 (± 0.03)	0–1
Black‐capped chickadee	309	0.85 (± 0.03)	0–2
Tufted titmouse	139	0.77 (± 0.04)	0–1
White‐breasted nuthatch	65	0.75 (± 0.05)	0–1
American goldfinch	80	1.65 (± 0.39)	0–30

**FIGURE 5:**
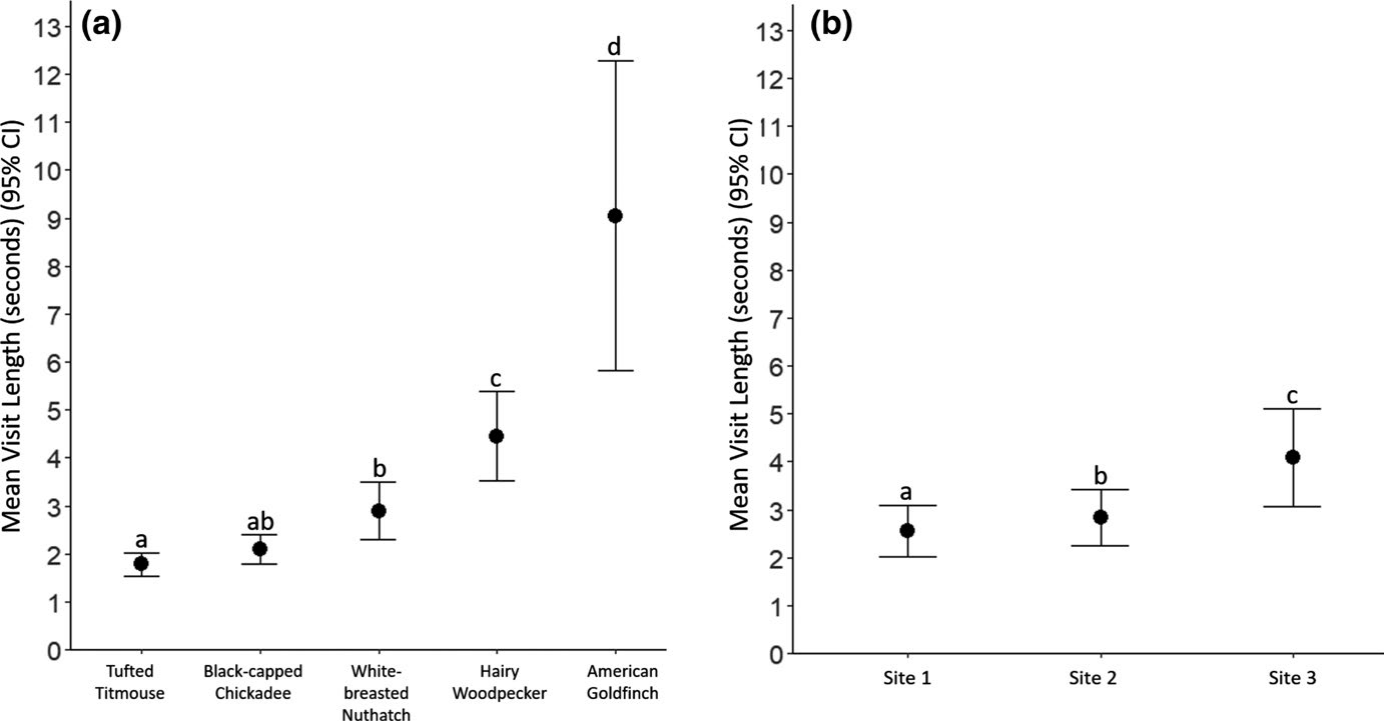
The raw mean length of visit at a feeder with 95% confidence intervals for (a) each species and (b) site. In both panels, letters indicate a statistically significant difference (*p* ≤ .05) in a post‐hoc pairwise comparison of the estimated marginal means (EMM) calculated from the model on the log‐scale. When calculating the EMM visit length for species, values were averaged over the levels of site, and when calculating the EMM visit length for sites, values were averaged over the levels of species

We found that visit length differed by species [*X* ^2^(4) = 158.14, *p* < .001], with American goldfinches visiting the longest and tufted titmice visiting the shortest (Figure 5). We also found that visit length differed by site [*X* ^2^(2) = 36.75, *p* < .001] (Figure 5).

## 4 DISCUSSION

### 4.1 RFID performance

Our research highlights the need to verify data gathered by automated systems. Our review of the relevant literature revealed that few ornithological studies using RFID include a description of data validation methodology and subsequent data quality. Researchers may verify their RFID data, but details of verification methods are often omitted or simply referenced as unpublished data (Schuett et al., 2017). We advocate that researchers take measures to verify and report evaluation data detailing the performance of their specific RFID system because accuracy will vary across system designs, locations, focal species, and other factors.

While we had downloaded data and used a PIT tag to check that our RFID system was working properly, we found that the system only detected 34% of all feeder visits by PIT‐tagged birds. In fact, most of the tagged birds had an average predicted probability of detection less than 0.50 (Figure 3), further demonstrating the importance of quantifying the detection probabilities of RFID systems. This detection rate is considerably lower than what is reported in the few RFID studies involving wild birds that verify the accuracy of their systems (100%, Falk et al., 2021; >99%, Firth et al., 2018; 74%, Zárybnická et al., 2016).

One factor likely contributing to our system's poor detection probability was the short visit duration of the tagged individuals. Each of the PIT‐tagged species visited the feeders for less than 3 s on average. Quick feeder visits were expected, as they have previously been reported for these and other species that take a single seed and consume or cache it elsewhere (Siekiera et al., 2020). Detection rates exceeding 99% have been achieved by RFID systems monitoring great tits *Parus major* (Firth et al., 2018), a quick‐moving species related to black‐capped chickadees and tufted titmice; so, short feeder visits do not universally restrict detection performance.

The physics of RFID field technology necessitates careful consideration and design of the receiving antenna. The low probability of detecting tagged birds by our RFID system was likely related to the design of the feeder and integrated antenna. The feeder port was always open, allowing birds access to seeds without ensuring that their PIT tag was read. Additionally, our video revealed that birds could land on parts of the platform and waterproof container not covered by the RFID antenna and still take seeds. Sufficient contact between tagged individuals and the antenna is necessary to collect accurate RFID data, and previous studies have used various approaches to achieve that contact. To reduce missed detections, feeders may restrict access to the food source until a bird's PIT tag is detected using a system similar to that described by Croston et al. (2016) or Ibarra et al. (2015). Alternatively, Bridge and Bonter's (2011) perch design mounts the RFID antenna on two short wooden dowels, requiring the birds to land on or within the antenna to access seeds.

Intra‐ and inter‐specific competition for access to the lone feeding port on our RFID‐enabled feeders may have also contributed to short visit lengths. Birds attempting to take seeds were often displaced by competitors vying for access to food. Displacement limited visit duration and the amount of time an individual's PIT tag was within range of the RFID antenna. Instead of one food access point, a feeder could have multiple RFID‐enabled ports, such as the feeder with two ports described by Moyers et al. (2018). Multiple feeder ports will not be necessary in all applications but may help to redistribute visits to allow for optimal data collection. Alternatively, Siekiera et al. (2020) describe a feeder equipped with a single RFID antenna with many “cells,” allowing the simultaneous recording of up to eight different birds accessing seed.

### 4.2 Characteristics of a visit

Different species exhibited different behavior while visiting the feeders. American goldfinch visits were longer than visits by the other four species, and video analysis revealed that the goldfinches behaved differently than the chickadees, titmice, nuthatches, and woodpeckers at the feeders. Goldfinches exhibited a “sit‐and‐eat” strategy, typically perching on the platform and consuming seeds there. In contrast, the other four species demonstrated a “grab‐and‐go” strategy, typically taking a single seed after landing on the platform and then leaving. This difference in strategies could be due to differences in the species' bill morphology. Chickadees, titmice, and nuthatches have bills that are not suitable for quickly dehusking sunflower seeds, so the birds may take a seed to a branch where they can better manipulate and exert the necessary force to open it (Soobramoney & Perrin, 2007). The bill of the American goldfinch is well‐adapted for dehulling sunflower seeds, so they were able to consume more seeds without leaving the feeder. Alternatively, leaving the feeder with a single seed may indicate that the food item is being cached for future consumption. Chickadees, titmice, and nuthatches are known to cache seeds, and sunflower seeds may be preferable for caching because their hulls could facilitate long‐term storage (Johansen et al., 2014).

Another potential explanation for the “grab‐and‐go” behavior is to reduce predation risk. Lima (1985) found that black‐capped chickadees tended to take a seed to cover when predation risk was higher but tended to stay at the food source when risk of predation was lower. Quick visits by all species may have been driven by perceived predation risk at the feeders, which can cause birds to increase their food consumption rate (Carrascal & Alonso, 2006). Even if risk of predation was not high, both inter‐ and intra‐specific competition for access to the feeder platform may have encouraged birds to relocate to a branch to consume their seed.

The presence of the camera near the feeder could have also affected the duration of a visit because of neophobia, or the fear demonstrated in response to novel stimuli (Greenberg, 2003), regardless of species. Chickadees, titmice, and nuthatches are all known to exhibit neophobia when novel objects are introduced near feeders (Stanback & Burke, 2020), and we observed these species to be apprehensive about landing on the feeder platforms when the camera was first deployed. All three tagged species demonstrated aversion to the camera by landing on the platform and immediately leaving after looking directly at the GoPro case. Eliminating the first video segment of each recording period from our analyses limited the likelihood of observing behaviors altered in response to the camera's introduction to the site. Neophobia still may have influenced behavior in some of the video segments; however, we observed that the birds quickly became habituated to the GoPro, apparently ignoring it or occasionally perching on it as a staging point before moving to the feeder. Habituation is a reduction in response over multiple exposures to a neutral stimulus, and it is a process that may be accelerated by offering a supplemental food source—such as seed feeders—to attract birds near the stimulus, in this case the GoPro (Knight, 2009; Whittaker & Knight, 1998). Future studies could further limit potential effects of neophobia by attaching a permanent camera mount and GoPro case to the feeding system. The constant presence of a feeder‐mounted camera fixture would be more conducive to habituation, as it allows additional time for the birds to become familiar with the hardware rather than introducing and removing the stimulus each week.

Despite the limited time spent at feeders by all five species, individuals were mostly successful in consuming or leaving with at least one seed per visit. In 79.4% of visits by all five species, a seed was either consumed at the feeder or taken by the bird. Bridge and Bonter (2011) reported a success rate of 81.1% for black‐capped chickadees; in our study, black‐capped chickadees had a similar foraging success rate of 79.0%. A high foraging success rate is noteworthy given the high nutritional value of the black‐oil sunflower seeds supplied by the feeders. Black‐capped chickadees, tufted titmice, and white‐breasted nuthatches are known to prefer black‐oil sunflower seed (Horn et al., 2014), a food that is high in fat and an important source of energy. Given the short visit duration and the high energy food reward, birds were likely gaining a high return on their energetic investment in foraging.

## 5 CONCLUSIONS

When using autonomous data collection systems like RFID, it is imperative that researchers do not rely exclusively on the technology. Technology provides many benefits, but it is not perfect, and that imperfection needs to be quantified and communicated where possible using appropriate validation methods. Including a procedure for validating our data revealed that our RFID system failed to consistently record the study's events of interest: visits to a supplemental feeder. Because of the unreliability and lack of consistency in the data collected, therefore, conclusions drawn from analysis of the RFID data would have been inaccurate.

While our study specifically investigates the accuracy of RFID detection in ornithological applications, use of video for validation could be replicated in other fields that employ RFID tags to monitor animals. Video may also be used to determine the behavioral characteristics associated with RFID detections. The definition of an RFID “read” is not universal and will change based on the study species, study system, and research question. Observing tagged organisms as they interact with an RFID reader can provide useful insight into the characteristics of an RFID read across many ecological applications of the technology.

## CONFLICT OF INTEREST

The authors declare no competing interests.

## AUTHOR CONTRIBUTIONS

**Eric J. Hughes:** Conceptualization (lead); data curation (equal); formal analysis (supporting); investigation (lead); methodology (equal); project administration (equal); resources (equal); software (supporting); validation (equal); visualization (supporting); writing–original draft (lead); writing–review and editing (lead). **Rachael P. Mady:** Conceptualization (supporting); data curation (equal); formal analysis (lead); funding acquisition (lead); investigation (supporting); methodology (supporting); project administration (supporting); resources (equal); software (lead); supervision (supporting); validation (equal); visualization (lead); writing–original draft (supporting); writing–review and editing (supporting). **David N. Bonter:** Conceptualization (supporting); methodology (supporting); project administration (supporting); resources (supporting); supervision (lead); writing–review and editing (supporting).

## Data Availability

The data supporting the findings of this study are openly available in Mendeley Data at https://doi.org/10.17632/9mnvpv2wcb.1.
